# Exploring barriers of health literacy on non-communicable disease prevention and care among patients in north wollo zone public hospitals; Northeast, Ethiopia, 2023: application of socio-ecological model

**DOI:** 10.1186/s12889-024-18524-8

**Published:** 2024-04-06

**Authors:** Eneyew Talie Fenta, Atitegeb Abera kidie, Misganaw Guadie Tiruneh, Tadele Fentabel Anagaw, Eyob ketema Bogale, Amanuel Addisu Dessie, Nigus Kassie worku, Mastewal Giza Amera, Hiwot Tesfa, Liknaw Workie Limenh, Amare Mebrate Delie, Birtukan Gizachew Ayal

**Affiliations:** 1 Department of Public Health, College of Medicine and Health Sciences, Injibara University, Injibara, Ethiopia; 2 Department of Public Health, College of Medicine and Health Sciences, Woldia University, Woldia, Ethiopia; 3 Department of Health Systems and Policy, Institute of Public Health, College of Medicine and Health Sciences, University of Gondar, Gondar, Ethiopia; 4 Department of Health Promotion and Behavioral Science, School of Public Health, College of Medicine and Health Sciences, Bahir Dar University, Bahir Dar, Ethiopia; 5 Department of Public Health, College of Medicine and Health Science, Dire Dawa University, Dire Dawa, Ethiopia; 6 Department of Pharmaceutics, University of Gondar, Gondar, Ethiopia

**Keywords:** Health literacy, Socio ecological model, Non-communicable disease, Ethiopia

## Abstract

**Background:**

Health literacy is the important for the prevention of non-communicable disease to make informed health decisions, and practice healthy and protective behaviours. Therefore, application of socioecological model to this study aimed to identify multilevel factors on health literacy among patients and develop scientific health communication interventional strategies to improve health literacy on non-communicable disease prevention and care.

**Objective:**

To explore barriers of health literacy on non-communicable disease prevention and care among patients in north wollo zone public Hospitals, Northeast Ethiopia, 2023.

**Method:**

In this study phenomenological study design was conducted from February 5 to 30/2023.We have used purposive sampling technique to select study participants from chronic follow up clinics. Data were collected using in-depth interview and focused group discussion in which audio was recorded, transcribed verbatim and translated to English. Thematic analysis was performed with atlas ti. 7 software.

**Result:**

In this study four main themes with seven subthemes were developed. The main themes were factors at the organizational, community, interpersonal, and intra-personal factors. The poor knowledge, lack of enough money for transportation and medication at the hospital were identified as barrier to get early diagnosis and treatment. Some participants explored that they have no any support from family or others. The cultural norms like weeding and funeral ceremonies enforce patients to consume prohibited substances like alcohol and salty foods.

**Conclusion:**

In this study different barriers of health literacy were explored. Lack of knowledge, economic problems, lack of social support, poor communication with health care providers, cultural influences, lack of regular health education, lack of access to health care services and poor infrastructure were main barriers of health literacy in patients with NCD. Therefore, we recommended all concerned bodies to work on social and behavioral change communication intervention focusing on awareness creation, supply of drugs and create supportive environment to get accessible and affordable health care service to decrease the impact of non-communicable disease at personal, community and national level.

## Introduction

Non -communicable diseases (NCD), such as cardiovascular diseases, cancer, diabetes, chronic respiratory diseases are the leading global cause of death and are responsible for over 71% of deaths worldwide, with 85% of these occurring in developing countries. These NCDs share key modifiable behavioural risk factors like tobacco use, unhealthy diet, lack of physical activity, and the harmful use of alcohol, which in turn lead to overweight and obesity, raised blood pressure, and raised cholesterol, and ultimately disease [1, 2]. World health organization ( WHO) developed an action plan from 2013–2020 to address population based risk factors and integrated management of NCDs at the primary health care level and, in Ethiopia 42% deaths and 27% are premature deaths before 70 age is cause by NCDs [3, 4].

Health literacy (HL) is the degree to which an individual have the capacity to obtain,process and understand basic health information’s to make appropriate health decisions [5, 6]. WHO positioning HL as one of pillars for achieving sustainable development and health equity [7]. HL is used to get reliable information, and empowers people to make informed health decisions, practice healthy and protective behaviours [8, 9]. The social ecological model (SEM) targets five levels of influence for health related behaviours which are: Intrapersonal factors, Institutional; community factors; and finally public policy level factors [10].

It has been shown that HL levels was lower among those who had low social status, have poor education and income levels, older population, gender groups, the level of medication adherence, higher BMI, increased systolic blood pressure, poor financial status, being, unemployment or retired status, poverty, and having a history of smoking or a history of consuming alcohol has association with low health literacy [11–13]. High level of health literacy is associated with improved preventive care, early detection of diseases, ability to access health care and management of chronic disease, use proper nutrition, avoidance of smoking and good medication adherence and also it shapes people’s health and the safety and quality of health care [14].

The studies also showed that structural barriers, such as insurance, transportation issues and limited information with in communities affect health literacy [15, 16]. Another study conducted in United Kingdom revealed that health literacy had association with lack of access to the Internet [17]. The study in Myanmar showed that watching medical-related TV series, accessibility to education were detected as significant determinants of health literacy. Cultural beliefs may also impact communication between patients and providers and affect a patient’s ability to follow a physician’s instructions [16, 18–20].

Health literacy is a social determinant of health, and poor HL is associated with poor education, poverty, unemployment, and low socioeconomic status, yet those with higher levels of education and income can have low health literacy. Studies have revealed that people with poor HL had lower health outcomes, and higher costs, and people of all ages, races, income levels, and educational levels are affected. Good vaccine HL is important to alter societal norms in promoting vaccine uptake and establishing a foundation for herd immunity at a level appropriate for each individual's age, mental capacity, gender, and environment [21–25].

The studies revealed that HL should improve especially in adult and elderly population, in those with a lower level of education for better disease prevention and adequate health literacy improved health outcomes and efficacy among older patients with chronic diseases [26, 27]. Health literacy interventions for chronic diseases include a range of strategies, such as education, self-management training, counselling, consultations, and other techniques, that are intended to improve the health-related knowledge, attitudes, abilities, and behaviours of people living with chronic illnesses [28, 29].

Even though, there are studies done regarding health literacy on non-communicable disease, that could not focus on exploring health literacy qualitatively for better interventional strategy development and the approach is not guided by the use of SEM to identify the levels of interventions. The application of social ecological model in health literacy could lead to more sustainable changes over time by creating supportive environments for people as they access and seek to understand health information, interact with health professionals, and move through their community and organizational contexts. Therefore, the application of socio ecological model to this study identified multilevel factors and provide information to develop scientific health communication interventional strategies to improve health literacy among patients in non-communicable disease.

## Methods and materials

### Study design, area and period

North Wollo is located in Amhara region with the capital city of Woldia found 521 km away from Addis Ababa and 360 km from Bahirdar. According to the 2007 census conducted by central statistics agency of Ethiopia it has a total population of 1,500,303. There are six governmental hospitals in North Wollo. These are Woldia Hospital, Kobo Hospital, Lalibela Hospital, Mekiet Hospital, Wadila Hospital, and Mersa Hospital. Institution based Phenomenological study design was used to explore barriers of health literacy among chronic patients in chronic care follow up clinics, north wollo zone three public hospitals, Northeast Ethiopia from February 5–30/2023 with the use of socio ecological model. The approach helps to describe particular phenomena, or the appearance of things, as lived experience and find meaning or is used in cases about which there is little knowledge available. Phenomenological study assists in looking at change processes over time, adjusting to new issues and ideas as they emerge among chronic disease patients in selected hospitals [30, 31].

### Study population and sampling procedure

The study population were all adult chronic patients receiving follow-up care in three selected public hospitals of north wollo. All chronic patients age above 18 years from selected public hospital were included in this study whereas participant who were seriously ill or not able to communicate, required emergency care, who had mental or cognitive impairment in the data collection period were not include in the study. In this study twenty-six in-depth interviews with 3 focus group discussions were conducted. A total of 22 individuals with chronic diseases follow up were participated in FGD. The participants were 9 DM patients (kobo 4, Meket 3, woldia 2), 11 HTN (5 kobo,2 Meket, 4 woldia), 3 Epilepsy (2 meket,1 kobo), 3 CHF patients (woldia 2, Meket 1). Three hospital managers, and 7 health care providers who work in chronic follow up clinic were participated for key informant interview. Purposive sampling was used in phenomenology; which focuses on particular characteristics of a population, which would be enabled to answer this research questions. Heterogonous purposive sampling was used to select people who had experienced the phenomenon [32].

### Data collection tool and procedure

The data was collected through face-to-face In-depth interview, key informant interview, and focus group discussion by experienced 4 masters holder health professionals. IDI, KII, and FGD guides were drafted through reviewing different related literatures. Written informed consent was obtained from each study participants. Each interview was audiotaped using digital voice recorder and filed notes were taken. The English version semi-structured open-ended question and were translated in to Amharic and again translated back to English. The length of the interview is guided by the process of information saturation, when the narratives become repetitive and no new data is revealed which lasts from 9 to 18 min for in-depth interview and 1 h to 1:40 h for FGD for patients, and health care and hospital manager key informant interview lasts 8 to 23 min.

### Data quality control

To maintain the credibility of the research findings, the study participants was observed persistently at the time of the interview. Peer-debriefing was done for the questioner and transcripts was given to my colleagues. Member checking was conducted by returning the preliminary findings to some participants to correct errors and challenge what they perceive as wrong interpretations. Dependability was attained through accurate documentation by minimizing spelling errors through frequently observing data and including all documents in the final report, such as including the notes written during the interview and ensuring that the details of the procedures was described to allow the readers to see the basis upon which conclusions was made. The data analysis, interpretations, and conclusions were continuously peer reviewed [33, 34].

To achieve confirmability of the study findings: raw data, interview and observation notes, documents and records collected from the field, and others was documented for crosschecking and to conduct an audit trial, where triangulation was used. To maintain the transferability of the finding, appropriate probes was used to obtain detailed information on responses, and study participants was selected based on their specific purpose to answer study questions and to get greater in-depth findings.

### Data processing and analysis

We have used consolidated Criteria for Reporting Qualitative Research (COREQ), a 32-item checklist that is used to report important aspects of the research team, study methods, context of the study, findings, analysis and interpretations. After data collection, the investigators transcribing the audio-record data in to Amharic, local language, translated into English, and then read and re-read the several times to code the data, to detect emerging themes and sub-themes. The translated document was coded line-by-line and grouped into themes based on the concepts they contain. Responses was categorized under each theme and sub-theme. Interpretations of the qualitative data was dependent upon patients’ descriptions of their experiences and perceptions, which the researchers check against the verbatim transcripts for accuracy and consistency.

Lastly, investigators interpret the theme to reveal core meanings of the experiences and presenting the discoveries of the study specifically, thematic analysis technique was used to analysed the data. Quotes was used to highlight each category and show association with each theme. The principal investigator reads and re-read the transcriptions several times to coding the data, then 32 categories were extracted from 128 codes by combination similar meaning then 7 sub-themes 4 major themes were identifying, and thematic approach was used for analysis, which could emphasize identifying, analyzing, and interpreting patterns of meaning [35].

## Result

### Socio-demographic information of the participants

In this study ten key informant interview, twenty-six in-depth interviews with 3 focus group discussions were conducted. A total of 22 individuals with chronic diseases follow up were participated in FGD. The most frequently reported diseases were hypertension, diabetes, chronic heart disease and epilepsy. The participants were 9 DM patients, 11 HTN, 3 Epilepsy 3 CHF patients totally 26 in-depth interviews were conducted. Also 22 FGD were conducted in three hospitals from Meket hospital,7 chronic disease patients (3 HTN, DM, bronchial asthma, Epilepsy, CHF 1 each) were participated. In woldia referral hospital totally 6 participants (3 HTN, 2 DM, and 1 CHF), in kobo hospital 9 NCD patients (DM4, HTN 3, Bronchial asthma 1, and 1 CHF) were included in the study. For key informant interview 4 medical doctors and 6 nurse professionals were participated (Tables 1 and 2).

**Table 1:** Socio demographic information on in-depth interviews on exploring barriers of health Literacy on non-communicable disease prevention and care among patients in north wollo zone public hospitals; Northeast, Ethiopia, 2023: application of socio-ecological model

Characteristics		Frequency
Age	18–39 years	5
	40–49 years	3
	50 years and above	18
Sex	Male	15
	Female	11
Educational status	No formal education	17
	Primary education	6
	Secondary and above	3
Marital status	Married	21
	Divorced	2
	Widowed	3
Residence	Rural	15
	Urban	11
Type of chronic disease	DM	9
	HTN	11
	Epilepsy	3
	CHF	3
	Total	26
Duration of with the disease	1- 5 years	11
	6–10 years	7
	11 years and above	8
	Total	26

**Table 2:** Summary of social demographic characteristics of the FGD participants on exploring barriers of health Literacy on non-communicable disease prevention and care among patients in north wollo zone public hospitals; Northeast, Ethiopia, 2023: application of socio-ecological model

Characteristics		Frequency
Total participants		22
Total focus groups		03
Age	15–39 years	7
	40–49 years	6
	50 years and above	09
Sex	Male	14
	Female	08
Educational status	Un educated	13
	Primary education	9
Marital status	Married	15
	Divorced	3
	Widowed	4
Duration of with the disease	1- 5 times	11
	6–10 times	6
	11 and above	5

### Thematic findings

**Fig. 1:**
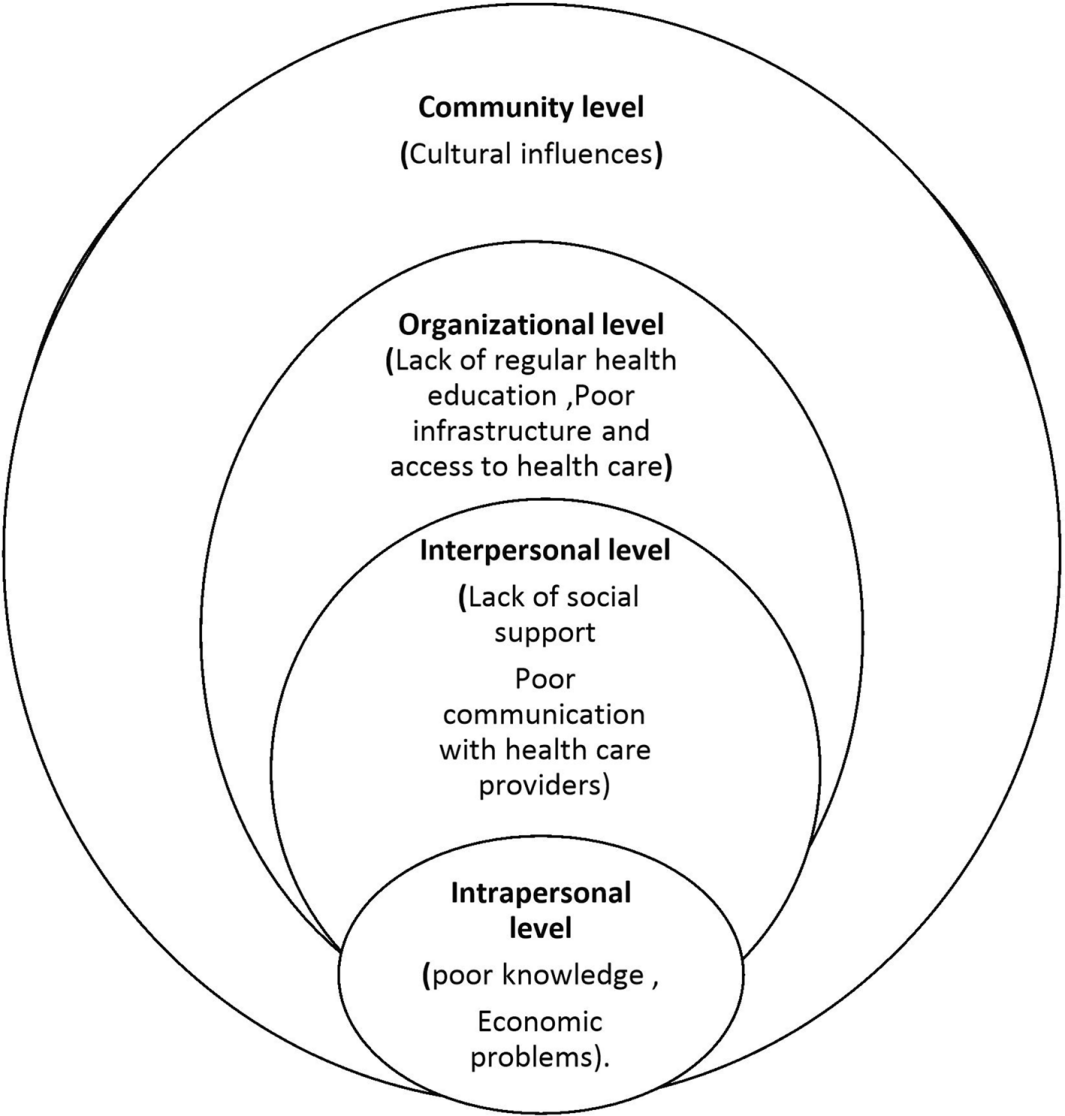
Representation of the key themes emerged from study participants

The findings that emerged from the analysis of the in-depth interview and focus group discussion were presented and arranged as major themes, sub-themes and categories. There are four (4) main themes and seven (7) subthemes (Fig. 1).

#### Theme 1: intra-personal (patient level) factors

In this study most of the study participants described that lack of formal education, poor knowledge on non-communicable disease, and economic problems were raised as the main barrier to health literacy on chronic disease. This theme has 2 subthemes, which are poor knowledge, and economic problems.

##### Poor knowledge

The study's participants talked about their inability to comprehend, their inability to get health information on non-communicable diseases, their lack of knowledge about their condition, their inability to take their medication, and their inability to adopt preventive measures for better disease control.

A 49 years old male epileptic patient having a chronic follow up for 10 years said that*: “I did not know how to take the medicine before which made it difficult for me to control the disease”.*

Health care providers also concluded that most patients don’t have awareness on the cause, treatment and prevention mechanisms of their disease before diagnosed for their diseases. This is explained as: A 27 years old male nurse working in the hospital for 4 years said that: “*Patients get information from health professionals. They know while they come to check themselves, most people with non-communicable diseases get information from a professional when they come for treatment. They have no awareness on their disease, almost 90% get information from health care professional*”.

Similarly, a 26 years old female doctor working for 2 years in NCD OPD explained that: *“Usually patients’ source of information on NCD is when they are told about the disease from the specialist. Occasionally they may have their own sources depending on their level of education, but usually from the professionals”.*

Regarding source of information, they heard about non-communicable disease from health care providers, using media like television or radio, other chronic disease patients, family, friends, and health care institutions. The following participant stated the same thing as:

*“Before I had screened the disease, I did not have any health information on my disease since I have no any education, and my source of information was from the community and television”. (61 years, male, Diabetic patient).*

##### Economic problems

The study participants reported that economic problems like, shortage of money for transportation to reach hospital, and to buy medication are barriers to early diagnosis and get treatment for their diseases.

*“I find it difficult to take more than one medication at once for my HIV, blood pressure, renal, and asthma. I also don't have access to the hospital pharmacy and don't have the money to buy all of my medications in private drug store”.* (60 years Female, FGD participant).

Another study participant explained as “*It is very difficult to buy the drug in private pharmacy. It is very expensive………*”. (48 years, Male diabetic patient participated in FGD).

A 26 years old female doctor working for 2 years in NCD OPD also expressed as: *“Most of the obstacles for controlling disease and using preventive methods in chronic patients is the economic problems. There may be edible foods for some diseases that their economy does not allow to get the proper nutrition for their disease…….”*

#### Theme two: interpersonal level factors

This theme has two subthemes which are lack of social support, and poor communication with health care providers.

##### Lack of social support

Even if most chronic disease patients have good family and social support some participants explored that they have no any support from family or others. The participants also explained that since no one remind them, they forget their appointment and taking medication regularly. The following participant stated the same thing as:

*“I am tired of taking the medicine, the lack of support from the family, government, and the lack of money has become a severe problem for me”.* (61 years male, DM patient).

Another 49 years male, epileptic patient having a chronic follow up for 10 years explained that: “*It's hard for me to come, because of I am alone I’m afraid to fail, I used to forget my follow-up appointment. Now I come to hospital by calling”.*

In addition to this lack of social support, absence of functional patients’ association like diabetes association, is a barrier for patients to control and care their diseases. As explained by male NCD focal nurse having greater than 12 years’ work experience:* “*Currently, *there is also a recently established Diabetes Association but not going as expected. Even though its role is to support our patients by some medicines especially for young (*< *25 years of age), it is intermittent and should be strengthen*”.

- Poor communication with health care providers

The study participants discussed that even if health care professionals provide health information about their disease status, they didn’t listen carefully, understand their need and give time to ask questions and get feedback for their disease.

Hypertensive, Female, 60 years, patient with one year duration described as:* “The health care professionals don’t give fair service to all equally, they don’t give enough health information for my disease, it is better if they give full information on the disease and then written medicine information than oral”.*

Another HIV and hypertensive female participant reported as: *“Since health care providers are busy, they did not care about me, except prescribing medication in my follow up, did not want to listen my complain and give advice by giving time is the main problem that I have to tell you”.*

#### Theme three: community level factors

##### Cultural influences

Even though, most of the participants are committed to take their medication timely and take the recommended prevention practices, some participants explored that cultural traditions with in the family and social ceremonies enforce them to drink and eat the forbidden things that worsen their health condition. Another participant also described that when there were special ceremonies around their home, they had forgotten to take their medication, not take timely, and use alcohol and other foods not allowed for NCD patients.

The study participant explained as

*“In our society, there are pressures to consume salty foods and alcoholic beverages at social events; there are occasional drug shortages; there is currently a shortage of laboratory resources; in my own negligence that I sometimes forget to take on time and at all”.* (77 years male DM patient).

Another female diabetic patient patient participated in FGD also said: *“……………. for example, when there was a wedding, I increased the amount of fat and sugar intake and my blood sugar became 400 in the last month. But now my blood sugar is 78 since I avoid taking these kinds of diet”.*

A 30 years old female nurse working for 5 years as BSC nurse also reported as *“There are social ceremonies that the people enforce patients to use the forbidden food and drink since most of the society did not understand the disease very well, for example, people around the hypertensive patient said that what is the problem if you eat salt for today…”.*

#### Theme four: organizational level factors

This theme has two subthemes, Lack of regular health education, poor infrastructure and access to health care were explored as barriers of health literacy on non- communicable disease prevention and care.

##### Lack of regular health education

The study participants reported that they had try to teach their patients about chronic disease when they come for appointment at clinics, but it is not continuous heath information provision by using different health education method like Television radio in most hospitals. The participant explained that they couldn’t organize drug information centre that can provide information for general education about medication on chronic diseases at the pharmacy level.

A 29 years old female working as a nurse for 4 years in the hospital reported as: “To increase patient’s knowledge, we provide health education for chronic patients at waiting area together. Still, we didn’t use media”.

32 years. Male participant nurse expressed as: *There are many written leaflets, so we distribute them to every patient who comes here, give health education, but did not use television; radio and did not keep the schedule. We also conduct a screening for hypertension and diabetes in clinics and at community level”.*

*“In the previous year, before patients enter to OPD health education was given 2 times a week. But now it has stopped, we prepare, and distribute leaflets, most of the patients were understand and apply what we said”.* (33 years old female, Nurse).

##### Poor infrastructure and access to health care

The study participants described that distance of home from the hospitals, and absence of comfortable roads to get hospital were the major obstacles in getting health information for early treatment*. male participant 54 years, diabetic and hypertensive patient explored as: “I travelled on foot from a remote rural area and arrived too late to take medication for my follow-up appointment”.* Similarly, male NCD focal nurse working for greater than12 years also expressed as: “*patients usually come from far place. So, they don’t come on time, especially diabetic patients who are expected to screen for fasting blood glucose before 8 h, are usually tested after 8 h and the result is not that much believable…. The cases are loaded and the hospital far for patients.”*

Almost all participants also described that the examination rooms were not comfortable to get health information and treatment appropriately. They explored that the buildings are narrow, no waiting area to get care and support easily.

A 27 years old male nurse working for 4 years said: *“The main problem is that our patients with non-communicable diseases have only one chronic OPD. So, since there are many NCD patients and the lack of class and trained professionals is a problem”.*

## Discussion

Health literacy includes all of the skills required to receive, interpret, and process basic health information and services in order to make significant health decisions [36, 37]. HL enhances individuals’ ability to participate in decision-making processes in various aspects of life concerning individual and community health [38, 39]. Our study identified four main themes with seven subthemes. These were patient level, Interpersonal level, Community level, and organizational level factors.

The participants explored poor knowledge on non-communicable disease, and economic problems were the main barrier to get health related information and health services for their chronic disease. This was comparable with study done in Nepal which stated that health literacy was affected by education, knowledge of health services and health problems, access to good quality information [40]. The study in Bangladesh supported that unawareness of the severity, lack of knowledge on NCDs and low health literacy were barriers to change in behaviour on chronic disease prevention and care [41]. Another study in Brazil described that poor and less accessible education, lower knowledge about the disease, and difficulties in understanding the medical instructions were factors associated with limited health literacy [42]. This might be lack of education leads to poor knowledge and understanding on NCD prevention and care.

The study's participants talked about that despite giving patients health information regarding their disease status, medical professionals didn't pay close attention, or allow enough time for their patients to ask questions. This is consistent with the study discussed that access to good quality information, communication skills of staff, health worker’s attitudes have effect on health literacy on non-communicable disease patients [40]. The communication between medical professionals and patients have an impact on the patients' knowledge, motivation, decision-making, participation [43]. Another study also described that medical mistrust was associated with poorer communication with providers and patients’ health literacy level [44]. The study in Australia revealed that inadequate understanding, poor support from family and friends, conflicting advice from and poor communication among specialists were barriers to get the recommended care for NCD patients [45]. The possible reason might be because of good communication with health care providers may help to get appropriate health information on NCD prevention.

The study's participants complained about how financial issues, such as a lack of enough money to travel to the hospital for treatment prohibit them from receiving an early diagnosis and treatment. This is comparable with other studies which reported that low social status, financial deprivation were barriers to health literacy [46, 47], and Low health literacy was associated with living in poverty, lacking consistent health insurance [48]. Another the studies showed that those groups more at risk of socioeconomic deprivation had low the health literacy status [49–51]. One plausible explanation could be that those with better financial standing find it easier to pay for the medications they are prescribed.

Even though most the study participants with chronic diseases have strong family and social networks, some participants mentioned that they don't have any support from their families or others. This was consistent with the studies which explains education, income, perceived health and social isolation have effect on health literacy [52, 53]. The studies discussed that social support in which the help of relatives and friends is important in assessing the information on chronic disease to increase health literacy of patients on their chronic disease [54, 55]. One explanation could be that patients who receive support from their families find it easier to remember when to follow up, and they may also be eligible for financial assistance to help pay for their medical expenses.

The participants explored that cultural norms enforce the consumption of prohibited substances at social events and family gatherings, which worsens their health. This is comparable with the study done in Kenya showed that culture and misinformation were determinates of health literacy on non-communicable disease [56] and the study in Germany revealed that family and peer influences within the different societies and cultures has effect on health literacy [57]. The studies also examined that cultural beliefs have impact on communication between patients and providers and affect a patient’s ability to follow a physician’s instructions [58–60]. This implies that cultural mis information on drinking and eating habits during social ceremony have impact on health literacy level of NCD patients.

In this study poor access of health care and poor infrastructure were the main barriers in getting care for chronic disease patients. This was consistent was the study in Bangladesh reported inadequate laboratory facilities, and logistics were barriers in chronic disease prevention and care [61] another study also examined infrastructure problems like bad road network, unreliable electricity supplies, living far from health centres were barriers to get NCD treatment [62]. The study in Nigeria also described that poor road and weak transportation infrastructure limit health education and awareness programs to communities [63]. This might be because good infrastructure and access to health care improves timely care for NCD patients.

The strength of this study was that the use of phenomenology study design with the application of socio ecological model that could explore multilevel barriers of health literacy on non-communicable disease prevention and care. These results cannot be automatically generalized to the entire population of patients with chronic non-communicable disease.

## Conclussion

The finding of this study showed barriers to health literacy on non-communicable disease prevention and care with four major themes and seven subthemes. These were patient level, interpersonal level, Community, and organizational level factors. Most of the study participants discussed that they have no awareness and knowledge on non-communicable disease prevention and care. The participants stated that lack knowledge, and economic problems were the main barrier to get health information and on their chronic disease. The study participants discussed that health care providers didn’t listen carefully, understand their need and give time to ask questions and get feedback for their disease, and some participants mentioned that they don't have any support from their families or others. The participants reported that poor access of health care, and poor infrastructure were the main barriers in getting care. The participants explored that cultural norms enforce the consumption of prohibited substances at social events and family gatherings, which worsens their health. Therefore, we recommended all the concerned bodies should work to avoid barriers of health literacy on chronic disease patients at each level of influence to decrease the impact of chronic disease at personal, community and national level.

## Data Availability

The datasets used and/or analysed during the current study available from the corresponding author on reasonable request.

## Abbreviations

WHO: World health Organization
NCD: Non communicable diseases
HL: Health literacy
HLL: Health literacy level
DM: Diabetes mellitus
CAD: Cerebrovascular disease
COPD: Chronic obstructive pulmonary disease
SEM: Social ecological model
